# Activation of extracorporeal membrane oxygenation: a therapeutic approach to be considered

**DOI:** 10.5935/0103-507X.20190053

**Published:** 2019

**Authors:** Isabel Araújo, Pedro Raul, Francisca Monteiro, Mágui Lobo, Marta Rodrigues, Filipe Fernandes

**Affiliations:** 1 Instituto Politécnico de Saúde do Norte, Escola Superior de Saúde do Vale do Ave, Cooperativa de Ensino Superior Politécnico e Universitário - Vila Nova de Famalicão, Portugual.; 2 Instituto de Investigação e Formação Avançada em Ciências e Tecnologias da Saúde, Cooperativa de Ensino Superior Politécnico e Universitário - Vila Nova de Famalicão, Portugal.; 3 Serviço de Urgência Médico-Cirúrgica, Centro Hospitalar do Médio Ave - Vila Nova de Famalicão, Portugual.; 4 Sistema Nacional de Saúde 24 horas Linha directa - Portugal.; 5 Hospital de Braga - Braga, Portugal.; 6 Programa de Pós-Graduação em Abordagem ao Doente Crítico, Instituto Politécnico de Saúde do Norte, Escola Superior de Saúde do Vale do Ave, Cooperativa de Ensino Superior Politécnico e Universitário - Vila Nova de Famalicão, Portugual.

**Keywords:** Extracorporeal membrane oxygenation, Heart arrest, Critical care, Ambulances, Emergencies

## Abstract

**Objective:**

To describe the epidemiological profile of victims of cardiac arrest assisted using a nontransporting emergency medical service vehicle and to determine whether these patients met the criteria for the use of extracorporeal membrane oxygenation.

**Methods:**

This study employed a retrospective, cohort, descriptive, and exploratory design. Data were collected in January 2018 in northern Portugal by consulting the records of nontransporting emergency medical service vehicles that provided assistance between 2012 and 2016. An observation grid was prepared that was supported by the instrument used for collecting data from the national registry of out-ofhospital cardiac arrests.

**Results:**

After applying the inclusion criteria, the sample consisted of 36 victims. Extracorporeal membrane oxygenation could have been applied to 24 victims during the period analyzed, which might have increased the odds for transplantation, survival, or both, for either the victim or other individuals.

**Conclusion:**

Nontransporting emergency medical service vehicles have the potential for inclusion in the extracorporeal membrane oxygenation network of the study area.

## INTRODUCTION

Currently, providing care to critically ill patients is a major challenge for health professionals. Recently increasing occurrences and complexity of health problems as well as the advances in technologies, for both diagnosis and treatment, have led to the need for updated knowledge and practices for the care management of critically ill patients.^(1)^

A critically ill patient is defined as "one whose survival depends on advanced means of monitoring and therapy because of the severe dysfunction or failure of one or more organs or systems." Thus, a critically ill patient is one whose life is threatened and depends on advanced means of monitoring, surveillance, and therapy.^(2)^ Different complications associated with critically ill patients can lead to cardiac arrest.

Cardiac arrest is defined as the cessation of the mechanical activity of the heart as confirmed by the lack of signs of blood flow caused by several clinical conditions. Cardiac arrest is characterized by four patterns of irregular heartbeat; ventricular fibrillation is the most common, followed by pulseless ventricular tachycardia, pulseless electrical activity, and asystole.^(3)^

Cardiopulmonary resuscitation maneuvers are the most common clinical intervention methods used to reestablish the spontaneous circulation of critically ill patients. Cardiopulmonary resuscitation should be an early, appropriate, coordinated, and standardized intervention for successful clinical reversal.^(4)^ It is possible to recover spontaneous circulation with basic life support, but this protocol is not sufficient in most situations; rather, advanced life support maneuvers are required, which consist of achieving the most appropriate return of spontaneous circulation through advanced medical care. Ideally, advanced life support must be started during the out-of-hospital phase and continue in the hospital. Whenever successful resuscitation occurs, it is essential to continue to provide postresuscitation care to preserve the integrity of the organs.^(5)^ Extracorporeal membrane oxygenation (ECMO) has already been introduced in certain countries for this purpose.

In Portugal, ECMO began as the consequence of the *influenza* A (H1N1) virus pandemic in 2009. Worldwide, numerous institutions have adopted the same type of intervention.^(6)^

Extracorporeal membrane oxygenation involves an extracorporeal circuit that directly oxygenates and removes carbon dioxide from the blood using an oxygenator. Gas exchange is performed via semipermeable membranes. The deoxygenated blood is drained into a drainage cannula using an external pump, which passes it through the oxygenator. The blood is then returned to the victim through a return cannula. When the blood is drained by the venous system and pumped into an artery, the circuit provides both respiratory and cardiac support.^(7)^

Extracorporeal membrane oxygenation is indicated to treat refractory cardiogenic shock in the presence of severe biventricular dysfunction, heart failure, cardiac arrest, or malignant ventricular arrhythmias.^(8)^ The mandatory criteria for the use of ECMO are a patient age between 18 and 60 years old; the absence of significant comorbidities; a shockable rhythm on first evaluation; evidence of pulmonary thromboembolism, hypothermia, or acute intoxication; cardiac arrest witnessed and promptly treated; a time from cardiac arrest to advanced life support of less than 10 minutes; a time from advanced life support to cannulation of between 10 and 30 minutes; and transport with a mechanical chest compression device during out-of-hospital cardiac arrest.^(9)^

Scientific evidence shows that the use of ECMO is important across different settings. ECMO reduces mortality rates and helps preserve organs for transplantation.^(10,11)^

In particular, the organization of responses to emergency situations has been the responsibility of the National Institute of Medical Emergency (*Instituto Nacional de Emergência Médica* - INEM). In the prehospital setting, clinical intervention aims to guarantee the population the provision of healthcare to injured victims and victims of sudden illness, ensuring the correct approach and stabilization of the victim at the site of the occurrence.^(12)^ One of the means of intervention in the prehospital setting is the use of a nontransporting emergency medical service vehicle, known in Portugal as *Viatura Médica de Emergência e Reanimação* (VMER). This prehospital intervention vehicle is intended for the rapid transport of a physician and a nurse with specific training in medical emergencies, particularly in advanced life support and advanced trauma life support.^(13)^

In light of the above, we asked the following research question: Do victims of cardiac arrest assisted by hospital's VMER meet the criteria for ECMO application?

Thus, this study aimed to describe the epidemiological profile of victims of cardiac arrest assisted by VMER and considered whether they met the criteria for the use of ECMO.

## METHODS

A retrospective, cohort, descriptive study was conducted. This study examined the victims assisted by the VMER of a hospital from a municipality located in the northern Portugal between 2012 and 2016.

During the study period, 8,330 victims were assisted; however, we selected only victims of cardiac arrest, resulting in a sample size of 1,525.

We defined the inclusion criteria as an age between 18 and 60 years; the absence of significant comorbidities; a shockable rhythm on first evaluation; evidence of pulmonary thromboembolism, hypothermia, or acute intoxication; cardiac arrest witnessed and promptly treated; a time from cardiac arrest to advanced life support of less than 10 minutes (may reach up to 15 minutes); a time from advanced life support to cannulation of between 10 and 30 minutes; and transport with a chest compression device during out-of-hospital cardiac arrest.^(9)^ After these criteria were applied, 36 victims suffering from cardiac arrest were included in a rational nonprobabilistic sample.

Data were collected by consulting the medical records. A grid of the records was created that organized the information into groups. The first group consisted of sociodemographic data (age, gender, year of occurrence, and history of previous diseases). The second group consisted of other variables (places of occurrence, type of occurrence, diagnostic hypothesis, actual time of occurrence, and heart rhythm). Finally, the third group referred to the inclusion criteria including the use of an automated external defibrillator (AED) on arrival; a time until VMER arrival of less than 15 minutes; and a distance between the place of occurrence and the emergency department of the reference center of less than 30 minutes.^(11)^

The study was performed with the authorization of the ethics committee and board of directors of the institution where the information was collected (Nursing Department of *Escola Superior de Saúde do Vale do Ave* (Vale do Ave Higher School of Health) - ESSVA/ENF-Va-017/2017). Respect for all deontological assumptions inherent in the ethics of human research were fully respected. The information collected was organized and subsequently analyzed using descriptive analyses (counts, means, percentages, and standard deviation) and inferential analyses (binomial test and chi-square goodness-of-fit test) using the *Statistical Package for the Social Sciences* (SPSS), version 23.

## RESULTS

The results are presented to first provide the sociodemographic characterization of the victims, followed by a description of the other variables.

### Sociodemographic characterization

A total of 36 victims suffering from cardiac arrest were included, 26 (72.2%) of whom were men. Patient age varied from 25 to 60 years old, with a mean age of 48.06 years and a standard deviation of 10.26 years. Regarding year of occurrence, 2013 had the highest rate of cardiac arrests (12 occurrences; 33.3%), followed by 2014 (9 occurrences; 25%), 2012 and 2016 (6 occurrences each; 16.7%), and 2015 (3 occurrences; 8.3%).

Mental illness and diabetes mellitus were the major previous diseases among women, followed by hypertension and respiratory problems; alcoholism and obesity were observed in only one victim. Among men, hypertension, diabetes mellitus, heart disease, obesity, and stroke were prevalent; mental illness, dyslipidemia, cancer, and liver disease as well as smoking and alcoholism were less frequent.

### Other variables

**Figure 1:**
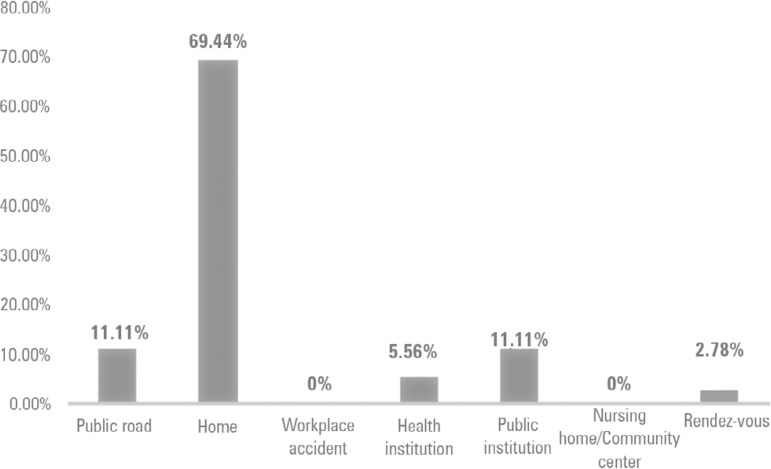
Location where victims were assisted by a *Viatura Médica de Emergência e Reanimação* in northern Portugal.

We present data on the place and type of occurrence, diagnostic hypotheses, real time of occurrence, and heart rhythm. Regarding the place of occurrence (Figure 1), the victim's home was the site where the VMER provided the most assistance (25 occurrences; 69.44%), followed by 4 occurrences (11.11%) on public roads, and 2 cases (5.56%) at public institutions. Assistance was provided at health institutions (health clinics) and in *rendez-vous* less frequently (2.78%).

**Figure 2:**
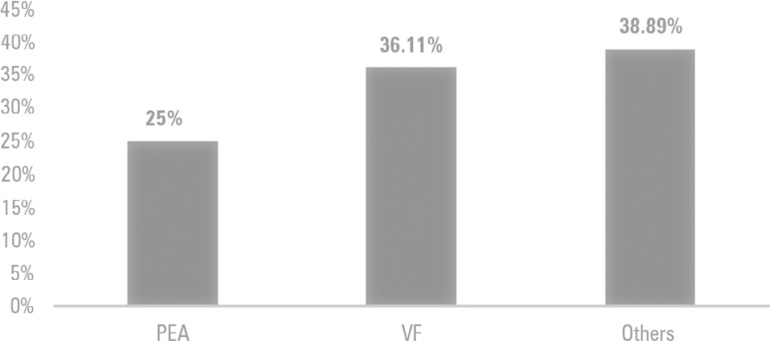
Heart rhythms on first evaluation of the victims assisted by a *Viatura Médica de Emergência e Reanimação* in northern Portugal. PEA - pulseless electrical activity; VF - ventricular fibrillation.

Regarding heart rhythm, those with asystole were excluded from the sample (Figure 2); the most common cardiac arrest rhythm on VMER arrival was ventricular fibrillation with 13 victims (36.11%), followed by pulseless electrical activity with 9 victims (25%). The remaining 14 victims (38.89%) did not exhibit cardiac arrest rhythm and were labeled as "other". These rhythms were evaluated on the arrival of the VMER to the site; in some cases, firemen had already begun basic life support maneuvers, and some victims had recovered their sinus rhythm. That is, on VMER arrival, the rhythms were not associated with cardiac arrest but were recorded as reversed cardiac arrest.

**Figure 3:**
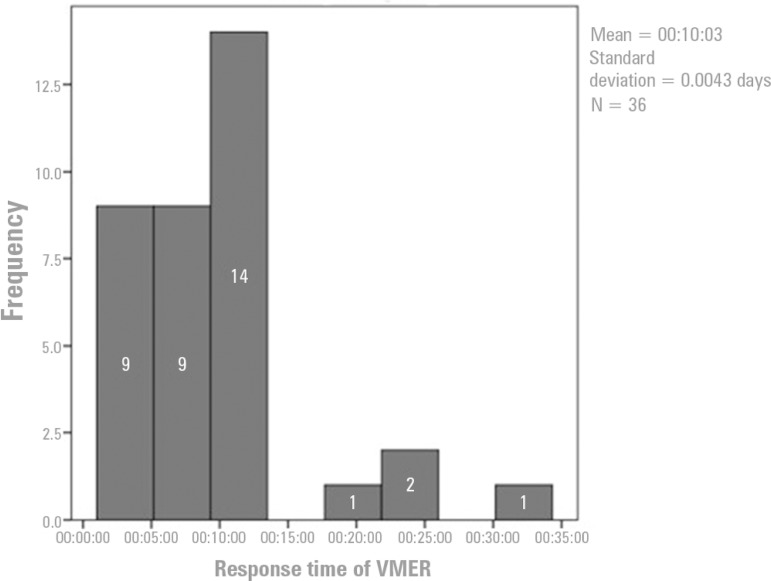
Response times to victims of cardiac arrest by *Viatura Médica de Emergência e Reanimação*. VMER - *Viatura Médica de Emergência e Reanimação*.

Figure 3 shows that the response time of the VMER (i.e., the time interval between the activation of the VMER and the arrival of the team to the site) varied between 1 and 31 minutes. The most common VMER response time ranged from 1 to 15 minutes (32 occurrences), followed by 15 to 31 minutes (4 occurrences). The average response time was 10 minutes 3 seconds, and the standard deviation was 0.0043 minutes. This value favored the survival of victims who met the ECMO criteria (supported in the optimized response window).

### Criteria for transplantation

Based on the analysis shown in table 1, which also displays the inclusion variables for transplantation, AED was applied to six victims (16.7%) and not used for 30 victims (83.3%). The time of arrest was less than 15 minutes in 32 occurrences (88.9%) and over 15 minutes in 4 occurrences (11.1%). Regarding the distance from the place of occurrence to the emergency department (a central hospital in northern Portugal), 24 patients (66.7%) were able to arrive at the hospital in less than 30 minutes, 11 (30.6%) exceeded the time limit of 30 minutes, and 1 (2.8%) provided no data regarding place of occurrence. To analyze these data, it was necessary to use the location information from the database provided by the VMER. The travel time to the emergency department of the central hospital in northern Portugal was calculated using Global Positioning System software.

**Table 1:** Univariate analysis of the inclusion variables regarding cardiac arrest victims for transplantation

Inclusion variables for transplantation	Options	Number of answers	%	Mean	Standard deviation
AED was applied upon arrival	True	6	16.7	-	-
	False	30	83.3	-	-
Time until VMER arrival was less than 15 minutes	True	32	88.9	-	-
	False	4	11.1	-	-
The distance from the place of occurrence to the emergency department was less than 30 minutes	True	24	66.7	-	-
	False	11	30.6	-	-
	No response	1	2.8	-	-

AED - automated external defibrillator; VMER - *Viatura Médica de Emergência e Reanimação*.

In the variable "year of occurrence", the value p = 0.165 and p > 0.05, it was accepted that there was not enough statistical evidence to state that the ocurrences were equally divided by the 5 years of occurrences. However, the evidence suggested that the cardiac arrests were not evenly distributed with regard to place of occurrence (p < 0.001 and p < 0.05; i.e., the numbers of arrests in each of the places were not the same because they more frequently occurred at home). The same result was found regarding heart rhythm (p < 0.001 and p < 0.05). Thus, the occurrences were not equally divided with regard to the different heart rhythms (Table 2).

**Table 2:** Inferential analysis: chi-square goodness-of-fit test for inclusion variables for transplantation and other variables

Chi-square goodness-of-fit test	Sigma (p)
Year of occurrence	0.165
Place of occurrence	0.000
Heart rhythms at first evaluation	0.000

## DISCUSSION

The main objective of this study was to identify whether victims of cardiac arrest assisted by a hospital VMER met the criteria for ECMO application. The study was conducted in northern Portugal and used data from a VMER database with a target population of 8,330 victims. The final sample was 36 patients after applying the inclusion criteria.

The mean age of these victims was 48 years old. Men predominated over women. This gender profile corroborates the results of Branco,^(14)^ who also observed a higher ratio of male victims. This profile might be related to the personal histories of the victims, where men show a predominance of hypertension, diabetes mellitus, stroke, heart failure, and smoking, whereas women show a predominance of mental illness. According to data from the National Institute of Statistics,^(15)^ mental and behavioral disorders are predominant in women. On the other hand, cardiovascular diseases predominate in males, which helps to understand the characteristics of the victims.

More VMER activations were observed in 2013. Cardiac arrest predominantly occurred at home, and no comparison data exist for these occurrences. Given that the age of the victims varied from 20 to 60 years, most VMER activations occurred after work hours and during lunch time because victims of this age range are not institutionalized.

The most prevalent heart rhythm was ventricular fibrillation, followed by pulseless electrical activity. The current data were identical to those of Branco,^(14)^ who showed that ventricular fibrillation was the second most prevalent rhythm, whereas asystole was the main cardiac arrest rhythm observed. However, we did not include the asystole rhythm in our results because its exclusion is one of the criteria for ECMO activation. Thus, 310 victims with asystole rhythm were excluded from the sample. According to the study, this variable was related to the time in which the victim was without basic life support between the activation time of the INEM and the consequent emergency team arrival at the location of the victim.

The VMER response time was less than 15 minutes in most cases. Therefore, we believe that this rapid response was crucial for the observation of victims with rhythms other than asystole. These data also indicate that it might be possible to apply ECMO because a response time of less than 30 minutes is an inclusion criterion. The rhythm variables were related to the various procedures in the approach to victims in cardiac arrest, namely automatic external defibrillation.

Automated external defibrillator was applied to some victims but not by the VMER; rather, this team uses a manual defibrillator.

The time from cardiac arrest to the beginning of the application of basic life support maneuvers was less than 15 minutes for most of the occurrences; however, basic life support was started after more than 15 minutes in some cases. Additionally, some cases did not list information concerning the beginning of cardiac arrest; thus, this parameter could not be calculated. Branco^(14)^ reported that basic life support was started no later than 10 minutes after cardiac arrest, ranging from 0 to 5 minutes.

A primary criterion for ECMO activation is a time of less than 30 minutes from the place of occurrence to the emergency department. In this study, 24 events met this criterion, but 11 exceeded the maximum limit of 30 minutes. Therefore, ECMO could have been applied to 24 victims during the period analyzed, which might have increased the odds of transplantation, survival, or both for either the victim or other individuals. Therefore, it is important the inclusion of VMER in the ECMO program to increase the possibility of victim recovery.

The development of this study involved certain limitations; thus, we suggest conducting additional studies on this topic. We were confronted with a scarcity of articles on the subject, a lack of records regarding the victims, and missing data regarding complication rates, survival rates, recovery rates, monetary costs, and professional training.

## CONCLUSION

This theme was pertinent given the assumption that more knowledge increases the individual's capacity to correctly evaluate and properly perform and implement treatment because this topic is in the early stages of research. Extracorporeal membrane oxygenation contributes to increased survival rates for different age groups and reduces mortality rates among victims of cardiogenic shock after acute myocardial infarction, cardiotomy, and (especially) cardiac arrest. Furthermore, it enables and contributes to the recovery of organs for transplantation, thereby decreasing the numbers of cadaver donations and interventions needed among chronic patients over the medium term. We confirmed the importance of including the nontransporting emergency medical service vehicles of a peripheral hospital as an integral part of an extracorporeal membrane oxygenation program because some victims meet the criteria for its application. These data are essential for obtaining health gains.
